# The Gendered Consequences of COVID-19 for Internal Migration

**DOI:** 10.1007/s11113-023-09809-8

**Published:** 2023-06-28

**Authors:** Valerie Mueller, Camila Páez-Bernal, Clark Gray, Karen Grépin

**Affiliations:** 1 School of Politics and Global Studies, Arizona State University, Tempe, USA; 2 International Food Policy Research Institute, Washington, DC USA; 3 Department of Geography, University of North Carolina, Chapel Hill, USA; 4 School of Public Health, University of Hong Kong, Hong Kong, China

**Keywords:** Migration, Gender, COVID-19, Africa

## Abstract

Scant evidence exists to identify the effects of the pandemic on migrant women and the unique barriers on employment they endure. We merge longitudinal data from mobile phone surveys with subnational data on COVID cases to examine whether women were left more immobile and vulnerable to health risks, relative to men, during the pandemic in Kenya and Nigeria. Each survey interviewed approximately 2000 men and women over three rounds (November 2020–January 2021, March–April 2021, November 2021–January 2022). Linear regression analysis reveals internal migrants are no more vulnerable to knowing someone in their network with COVID. Rather, rural migrant women in Kenya and Nigeria were less vulnerable to transmission through their network, perhaps related to the possible wealth accumulation from migration or acquired knowledge of averting health risks from previous destinations. Per capita exposure to COVID cases hinders the inter-regional migration of women in both countries. Exposure to an additional COVID case per 10,000 people resulted in a decline in women’s interregional migration by 6 and 2 percentage points in Kenya and Nigeria, respectively.

## Introduction

Internal migration remains an important livelihood and risk management strategy for rural households in low- and middle-income countries (LMICs) (Beegle et al., 2011; Hirvonen, 2016; Lagakos et al., 2020). Remittances offer households liquidity that can be used to smooth their consumption amidst economic shocks such as the pandemic (Vanwey, 2004; Halliday, 2006; Yang & Choi, 2007). Within the first year of the pandemic, 50% of Kenyan and Nigerian households were food insecure (Mueller et al., 2022). Opportunities to earn auxiliary income were obliterated by the presence of curfews, lockdowns, and temporary school closures (Hale et al., 2021). Without access to credit, internal migrants in Kenya (KNBS, 2022a, b) and Nigeria (Mueller & Lee, 2019) might have lost vital supplementary income. The extent pandemic policies influenced the capacity for migrants to work is crucial to inform targeting within existing poverty reduction programs (Gerard et al., 2020). Although social assistance was expanded in Kenya and Nigeria to encompass newly vulnerable households (Devereux, 2021), prior evaluations of such programs suggest that the benefits are context-specific and may not be uniformly realized across demographic groups (Perera et al., 2022).

There is scant evidence relating the pandemic consequences on women’s mobility, especially in Africa. An emerging literature in Asia corroborates supply- and demand-side constraints on women’s labor at the onset of the pandemic affected their ability to work (Agarwal, 2021; Desai et al., 2021; Seck et al., 2021). Women from agricultural communities often work in nearby towns or cities to supplement household income (De Haan et al., 2002; Amare et al., 2021). Migrant women from rural Kenya play a vital role in selling the commodities of farmers to cities following the harvest period and bartering the fruit collected by other women in the community (Kiteme, 1992; Frances, 2002). Fewer women in Nigeria move for economic reasons (in comparison to being motivated by schooling or marriage) in part due to divergent ethnic and religious beliefs around women’s freedom of movement (Mberu, 2005; Isiugo-Abanihe and IOM Nigeria, 2016; Mueller & Lee, 2019; Amare et al., 2021). However, in both contexts, women’s mobility might have been affected differentially as social norms presented them with obligations to supervise pupils at home and manage the increased burden of domestic work (Yaish et al., 2021; Farré et al., 2022; Zamberlan et al., 2022; Mueller et al., 2023).

The implications of the pandemic on the welfare of migrant women have also largely been ignored by the literature. Migrant health may have been compromised through the carrier mechanism (Cruz et al., 2020; Varkey et al., 2020; Gupta et al., 2021; Lee et al., 2021; Ramírez-Aldana et al., 2021; Valsecchi & Durante, 2021; Li & Ma, 2022), malnutrition (Smith & Wesselbaum, 2020), and precarity (Khan & Arokkiaraj, 2021). Migrant women were oftentimes more exposed to the virus as their work required more manually-intensive tasks and personal interaction with employees and clients (Arora & Majumder, 2021). Upon their return home, they also possessed less agency over protective measures and bargaining power within the household after losing their income (Arora & Majumder, 2021).

While these mechanisms highlight the additional risks temporary migrants face during the transition period, return migrants may also have acquired auxiliary knowledge and financial resources to protect their well-being. Return migrants may have been better informed about how to improve their practices around social distancing and reduce contagion through the insights learned from living in their previous destinations (Valsecchi & Durante, 2021). In addition, if return migrants tend to be wealthier than others in their community (Beegle et al., 2011; Hirvonen, 2016; Lagakos et al., 2020), they may have been able to lower their exposure by sparing themselves or their family members from unsafe employment conditions or investing in protective measures. Earlier work reveals migrants behave altruistically (Osili, 2007), especially women (De La Brière et al., 2002; Vanwey, 2004). Other studies have more directly linked receipt of remittances to increased expenditures on health care, corroborating tendencies for migrant income to be used to avert familial health risks (Catalina Amuedo-Dorantes & Pozo, 2011; Ambrosius & Cuecuecha, 2013).

We use data from longitudinal surveys conducted in Kenya and Nigeria to explore how African migrants were affected by the pandemic. Approximately 2000 men and women were surveyed in each country in November 2020–January 2021, including the administration of a migration history module to capture respondent moves prior to COVID-19. The same respondents were then interviewed in follow-up rounds to determine their current locations in March–April 2021 and November 2021–January 2022. We first examine whether migrants who moved prior to the pandemic were more vulnerable to COVID-19 risk, differentiating effects by sex and urbanicity. We next evaluate under what circumstances outbreaks affected the internal migration of women relative to men given the disparate effects pandemic policies had on women’s labor market participation (Contreras-Gonzalez et al., 2022; Mueller et al., 2023). In what follows, we motivate our main research questions by reviewing the literature on the determinants and impacts of migration in the context of health crises and highlight our contributions to this literature (Sect. 2). We then describe the primary data and the empirical strategy (Sect. 3). Discussions of the findings (Sect. 4), alternative modeling and robustness checks (Sect. 5), and data limitations (Sect. 6) follow. Section 7 concludes.

## Literature Review

Our two central research questions focus on how exposure to health risks and their implications for mobility depend on one’s identity and local conditions. We first draw on the public health literature to discuss the potential drivers of disparities in health risk between migrants and their neighboring peers at the time of COVID-19.

Migrants have been identified as a population which endures a unique set of health risks. Vulnerability to disease stems from disparities in exposure (moving from a place of low infection risk to high infection risk) and socioeconomic differences at the destination (Lienhardt, 2001; Mari et al., 2012). Impromptu housing arrangements introduced by lockdowns, the lack of sanitation in informal settlements, and the cramped conditions in living quarters provided by employers increases migrants’ overall risk of contracting infectious diseases relative to other populations (Cruz et al., 2020; Gupta et al., 2021; Khan & Arokkiaraj, 2021). Migrants additionally face greater threats to their health given their heightened levels of malnutrition and food insecurity (Smith & Wesselbaum, 2020).

Women experienced greater levels of economic precarity during the COVID-19 pandemic (Josephson et al., 2021; Bau et al., 2022). Migrant women possessed fewer opportunities in the manufacturing, retail and trade, and service sectors and were unable to work remotely. The lack of remote work options for migrant women was met with increasing risk of occupational hazards relative to employed, non-migrant women or men, as their activities required working in congested factories (Kabir et al., 2020) or interacting with customers in densely populated areas where disease was more widespread (de Diego-Cordero et al., 2022).

Although migrant women appear more vulnerable to health risks, they may be better informed about averting health risks through the knowledge acquired through their networks (Valsecchi & Durante, 2021) or better equipped to invest in protective measures (Beegle et al., 2011; Hirvonen, 2016; Lagakos et al., 2020). Previous research examining these two channels in non-pandemic contexts have been limited to Mexico. There, migrant women obtain more accurate health knowledge than their non-migrant counterparts (Hildebrandt & McKenzie, 2005), while migrant households are more capable of increasing health investments (Amuedo-Dorantes & Pozo, 2011) and minimizing debt when having a member in need of hospital treatment (Ambrosius & Cuecuecha, 2013). Ginsburg et al. (2022) corroborate both channels may be at play during the COVID-19 pandemic in Africa. Specifically, migrants in South Africa were more likely to adopt protective (i.e., wearing a mask, sanitizing, and washing hands) and social measures (i.e., avoiding crowds, social events, and going out of the house) relative to their non-migrant counterparts. Although they do not distinguish effects by the gender of the migrant, a greater proportion of the migrant population consists of women. They also illustrate that the impacts on behavior coincided with improved socio-economic outcomes of migrants relative to non-migrants.

Given preliminary evidence of the mitigation uses of migrant income, we next use the existing migration and health literatures to illustrate the extent localized risk, captured by per capita cumulative COVID-19 cases, may interfere with employment migration. In 2021, both governments of Kenya and Nigeria imposed nationwide curfews to reduce the spread of the virus and manage security issues (Hale et al., 2021). In areas facing greater per capita risk, we might expect the capacity of would-be migrants to be compromised in a few fundamental ways based on previous pandemics. First, enforcement of isolation protocols has typically been stringent in outbreak-afflicted areas (Tower et al., 2014). Thus, curfews in COVID-prevalent areas might have impinged on the ability of rural migrants to search for work in urban areas or travel to destinations of greater distance (Kumar et al., 2021). Second, workers in areas with greater rates of COVID-19 risk might experience lower labor productivity and asset wealth to finance migration. Infected workers have diminished capacity to work in activities that require physical exertion, and households often respond to constraints on labor by depleting their assets (Talman et al., 2013).

Norms around the intrahousehold allocation of labor may exacerbate the migration effects from the above constraints on the mobility of women relative to men. The pandemic literature corroborates that, as members of the household and community fall sick, the domestic and caregiving responsibilities are typically relegated to women (Talman et al., 2013). Women experienced additional hurdles (prior to our first-round survey) in Kenya and Nigeria as national school closures mandated women (often adolescent siblings and mothers) to supervise pupils at home (Mueller et al., 2023). Subsequent curfews and restrictions on movement (which ensued at the time of the second and third rounds of our survey) might have hindered women from outsourcing these tasks to older relatives and paid caregivers. Taken together, patterns of internal migration are likely to be affected in areas with a greater number of cumulative cases. We expect fewer people to travel long-distance for work and women, specifically, to face greater immobility relative to men.

To answer the second research question, we build on the nascent literature which studies how migration patterns were influenced by the COVID-19 pandemic. An emerging consensus is that the pandemic depressed migration to cities (Fielding & Ishikawa, 2021; Borsellino et al., 2022). In a study of nine African and Latin American countries, Bargain and Aminjonov (2021) show the declines are smaller in places with higher regional poverty. The authors argue that enforcement of social distancing may have been lower in places where workers rely on casual labor as a main income source and where job-related tasks cannot be fulfilled remotely. These findings intimate that the migration effects may be negligible in our Kenya and Nigeria case studies. In addition to contributing auxiliary evidence in Africa, we identify movers and non-movers and their locations over time, which allows us to distinguish the migration effects of economic disruptions and policies by mobility pattern (e.g., within-region versus across-region moves) and gender of the respondent unlike previous studies that use telecommunications data (Jia et al., 2020; Persson et al., 2021), Google reporting (Bargain & Aminjonov, 2021), or administrative data on interregional flows (Fielding & Ishikawa, 2021; Borsellino et al., 2022).

## Data

We collected panel data from men and women in Kenya and Nigeria across three rounds during the pandemic: Round 1 (November–January, 2020–2021), Round 2 (March–April 2021), and Round 3 (November–January 2021–2022). Given the challenges in conducting surveys in-person, performing phone interviews was a common option during the pandemic (Josephson et al., 2021; Chasukwa et al., 2022). Our sampling frame was based on a Rapid Digit Dial (RDD) framework, a frequently administered approach in public opinion research (Gallup, 2008). The name originates from the procedure to reach respondents, which entails enumerators randomly generating dialed numbers and interviewing respondents on the phone following their consent. In the pandemic context in LMICs, this way of reaching respondents was refined by purchasing phone numbers from third-party distributers and then randomly calling the list to formulate the sample (Dillon et al., 2021). We imposed additional eligibility requirements prior to a respondent being interviewed: (1) they were at least 18 years old; (2) they were willing to be surveyed in follow-up rounds and provided the pertinent contact information; and (3) they spoke one of the languages of the enumerators on the team. Additional details regarding the questionnaire and construction of the sampling weights used in the analysis are provided in Mueller et al. (2022).

Our sample consists of an unbalanced panel of 2002 and 1930 adults in Kenya and Nigeria respectively. Table A1 shows the number of individuals per country that were interviewed over one, two, and three rounds. Attrition rates are rather high. For example, only 66 (64)% of our baseline sample were surveyed at all three rounds in Kenya (Nigeria). We estimate two cross-sectional linear probability regressions of attrition using baseline individual and household characteristics as covariates to gauge which demographic groups are more inclined to exit the survey at different stages. The results from Table A2 indicate that women are more likely to exit the Nigeria survey by endline. Age affects attrition quite differently across contexts; older women are more likely to exit the survey in Nigeria and younger women are less likely to exit the survey in Kenya at Rounds 2 and 3. Finally, education and wealth seem to affect retention in the Nigerian sample. Those with a secondary and tertiary education are more likely remain in the sample by endline, but individuals at the upper tail of the wealth distribution are more likely to exit. To account for attrition in our inferences, our sampling weights include an attrition correction following the procedure used in Kastelic et al. (2020).

### Migration Amidst the Pandemic

Migration data were collected for 5333 and 5105 person-rounds in the two countries. Table A3 displays the distribution of the sample across rounds. We first aim to describe how migration might have changed for different sub-groups amid the pandemic. In Rounds 1 and 3, we asked the respondents for the location of their current residence. In Round 1, we also asked respondents their location of residence in 2018 and 2019 at the time of the interview month in Round 1 as a reference point. The information from Rounds 1 and 3 was used to create three time-variant binary migration outcomes, which respectively hold a value of one (i) if the person moved to another location in the last year; (ii) if the person moved to another location within the same county (Kenya)/state (Nigeria), and (iii) if the person moved to another location within another county (Kenya)/state (Nigeria). According to the statistics in Table 1, approximately 26% of the Kenyan sample and 18% of the Nigerian sample reported moving to another region in the previous year. Men move slightly more than women: 30(18)% of men move relative to 23(17)% of women in Kenya (Nigeria). Interregional moves dominate over within-regional moves. Twenty-one percent of Kenyan respondents move to another county compared to 5% of Kenyan respondents who move within the same county. Similarly, 12% of Nigerian respondents move to another state compared to 6% of Nigerian respondents who move within the same state. International migrants were removed from the sample, since they were a minute fraction and unlikely to be contactable in follow-up rounds.^1^

**Table 1:** Summary statistics

	Kenya			Nigeria		
	Total	Women	Men	Total	Women	Men
Migrate in the last 12 months	0.26	0.23	0.30	0.18	0.17	0.18
Migrate within state/county in the last 12 months	0.05	0.05	0.05	0.06	0.06	0.06
Migrate to another state/county in the last 12 months	0.21	0.18	0.24	0.12	0.12	0.12
Female	0.61	1.00	0.00	0.52	1.00	0.00
Married	0.48	0.47	0.51	0.45	0.49	0.41
Single	0.46	0.46	0.46	0.53	0.47	0.59
Widowed	0.03	0.03	0.01	0.01	0.02	0.00
Divorce or separated	0.03	0.04	0.02	0.01	0.01	0.00
Age 15–25	0.47	0.46	0.48	0.39	0.38	0.41
Age 26–35	0.29	0.29	0.28	0.33	0.34	0.33
Age 36–45	0.14	0.15	0.13	0.18	0.20	0.16
Age 46–55	0.07	0.05	0.08	0.06	0.05	0.07
Age 56–65	0.03	0.04	0.01	0.03	0.02	0.03
Age more than 65	0.00	0.00	0.00	0.01	0.01	0.01
Not formal education	0.00	0.00	0.00	0.02	0.02	0.03
Pre-Primary education	0.00	0.00	0.00	0.00	0.00	0.00
Primary education	0.16	0.18	0.13	0.05	0.04	0.05
Secondary education	0.41	0.39	0.43	0.35	0.36	0.35
Higher education	0.43	0.42	0.44	0.58	0.58	0.58
Number of young children	0.70	0.74	0.62	0.78	0.73	0.83
Wealth quintile 1	0.23	0.24	0.20	0.20	0.21	0.18
Wealth quintile 2	0.22	0.22	0.22	0.18	0.18	0.18
Wealth quintile 3	0.21	0.20	0.22	0.22	0.23	0.20
Wealth quintile 4	0.17	0.18	0.16	0.21	0.20	0.21
Wealth quintile 5	0.18	0.16	0.19	0.20	0.17	0.23
Rural	0.56	0.57	0.54	0.36	0.35	0.37
Year 2019	0.37	0.37	0.36	0.37	0.37	0.36
Year 2020	0.37	0.37	0.36	0.37	0.37	0.36
Year 2021	0.27	0.26	0.28	0.26	0.25	0.27
*N*	5333	3363	1970	5105	2922	2183

All explanatory variables are defined from the baseline survey except the year indicators. Sampling weights applied

### Main Regression Samples

We create two person-round samples from the longitudinal data to quantify the relationships between (i) prior migration and exposure to COVID-related health risks; and (ii) lagged exposure to COVID-related health risks and migration. The analytical sample used to address (i) comprises 4979 person-years in Kenya and 4774 person-years in Nigeria (Table A4). Tables A5 and A6 display the summary statistics for the outcomes and explanatory variables included in analysis by total observations, as well as stratified by sex, location, and the intersection between sex and location. Exposure to COVID-19 is captured by a time-varying binary outcome that was collected in each survey round that holds a value of one if the person indicated that they knew someone with the disease. Prior migration is a time-invariant explanatory variable that was collected in Round 1, which indicates whether the person resided in a different location in 2018–2019. We further differentiate the historical migration episodes by whether the person moved to another location within the same region (county for Kenya and state for Nigeria) or across regions. At Round 1, approximately 28% of Kenyans and 13% of Nigerians report living in a different location than in 2018–2019. Twenty-seven percent of Kenyans and 12% of Nigerians report knowing someone infected with COVID-19 at any given point in the study. These statistics highlight vast differences in historical migration and exposure to COVID-19.

A second sample used to analyze relationship ii) consists of 2977 and 2861 person-years in Kenya and Nigeria, respectively (Table A7). We merged the longitudinal data with two additional data sources. We first added a dataset assembled by Innovations for Poverty Action (IPA, the survey firm involved in the longitudinal study) which captures the number of COVID-19 new cases by date and region (county in Kenya, state for Kenya) over the study period. We next added regional population figures using the 2016 Kenya Integrated Household Budget Survey and Wave 4 of the General Household Panel Survey in Nigeria available through IPA. Both sources of data were used to create a lagged COVID-19 exposure variable, which represents the number of cumulative new cases since the last round per 10,000 people. Our analytical sample excludes men and women who were only surveyed in Round 1 and includes migration episodes that occur between Rounds 1 and 2 and 2 and 3. Table A8 provides summary statistics for the migration outcomes and the lagged COVID-19 case variable included in the analysis. The average number of cases per 10,000 people in a region was 16 in Kenya and 12 in Nigeria over the study period.

## Methods

We estimate two empirical models to answer the research questions of interest using the two samples described in Sect. 3.2. To examine the relationship between prior migration and exposure to COVID in own networks, we employ the following model:

1 $${K}_{ir}={\alpha }_{0}+{\alpha }_{r}+{\beta M}_{i}+{\delta X}_{i}+{\varepsilon }_{ir}, r=\left\{\mathrm{1,2},3\right\}$$

Equation (1) expresses the correlation $\beta$ between an individual *i* knowing someone with COVID-19 in their network during round *r* and whether they migrated from their 2018–2019 to Round 1 location, *M.* We evaluate the impact of migration broadly and then distinguish by migration within across regions. We further condition the relationship by survey round, ${\alpha }_{r}$ and baseline demographic characteristics (marital status, age, education, and the number of children in the household), *X.* Standard errors are clustered at the regional level (county for Kenya, state for Nigeria). Our first hypothesis is that migrants may be less vulnerable to COVID through their own networks, or $\beta <0$. We estimate the model restricting the sample by sex and location to recognize heterogeneity in effects by migrant characteristics and population density. The migration literature suggests that the effects may be greater in magnitude for women due to evidence that they tend to be altruistic relative to men, contribute toward health investments, and acquire improved health knowledge from destination networks.

We next apply a second model to establish the association between lagged (*r-1*) COVID cases *C* in location *j* and migration since the last round (between Rounds 1 and 2, and Rounds 2 and 3), *M* (all moves, within region, and across regions) in round *r*:

2 $${M}_{ijr }={\alpha }_{0}+{\alpha }_{i}+{\alpha }_{r}+{\lambda C}_{jr- 1}+{\varepsilon }_{ijr}, r=\left\{\mathrm{2,3}\right\}$$

We leverage the fact that the outcome and explanatory variable of interest vary by individual over time to estimate a fixed effect model, including individual ${\alpha }_{i}$ and round ${\alpha }_{r}$ fixed effects in (2). Standard errors continue to be clustered at the regional level. Although (2) controls for time-invariant and time-specific factors that might influence migration, the exclusion of time-varying factors interferes with our ability to causally identify $\lambda$. We therefore rely on the estimated correlation of $\lambda$ to assess whether exposure to the pandemic restricts mobility, especially among women. Our second hypothesis implies $\lambda <0$, particularly when restricting the focus to the sample of women.

## Results

Tables 2 and 3 provide the estimates of the parameters and standard errors for model (1) using data from Kenya and Nigeria, respectively. There is no apparent relationship between prior migration and knowledge of someone with COVID-19 in Kenya, when focusing on an aggregate measure of migration (Table 2). However, rural women that migrated prior to Round 1 are 9 percentage points less likely to know someone with COVID-19 Nigeria (Table 3). Our findings in Nigeria are consistent with earlier work in Mexico, where improvements in child health were driven by wealth effects and increases in health knowledge among women’s migrant networks (Hildebrandt & McKenzie, 2005).

**Table 2:** Determinants of knowledge of someone With COVID-19 for national migration, Kenya

Variables	(1)	(2)	(3)	(4)	(5)	(6)
	Women	Rural women	Urban women	Men	Rural men	Urban men
Migrated from 2018 to 2019 to Round 1 location	0.047	0.062	0.035	0.011	0.087	− 0.085
	(0.044)	(0.056)	(0.060)	(0.045)	(0.055)	(0.075)
Single	0.092***	0.143***	0.026	0.012	0.041	0.010
	(0.033)	(0.039)	(0.055)	(0.056)	(0.061)	(0.114)
Widowed	0.078	− 0.096	0.256**	− 0.521***	− 0.334***	− 0.705***
	(0.102)	(0.081)	(0.112)	(0.154)	(0.105)	(0.216)
Separated–divorced	− 0.059	− 0.091	− 0.026	0.133	0.262	0.097
	(0.108)	(0.116)	(0.143)	(0.136)	(0.180)	(0.142)
Age 26–35	0.163***	0.173***	0.136**	0.155**	0.173***	0.168*
	(0.041)	(0.038)	(0.069)	(0.063)	(0.065)	(0.102)
Age 36–45	0.389***	0.359***	0.432***	0.333***	0.269***	0.398***
	(0.053)	(0.049)	(0.090)	(0.093)	(0.083)	(0.150)
Age 46–55	0.386***	0.464***	0.364***	0.256***	0.173**	0.349**
	(0.057)	(0.063)	(0.093)	(0.084)	(0.085)	(0.138)
Age 56–65	0.597***	0.475***	0.715***	0.320**	0.429***	0.207
	(0.081)	(0.101)	(0.067)	(0.132)	(0.152)	(0.179)
More than 65	0.293**	0.434***	0.196	0.342	0.131	0.607
	(0.115)	(0.135)	(0.125)	(0.260)	(0.123)	(0.481)
Secondary education	0.110***	0.096**	0.143*	0.165***	0.107*	0.257***
	(0.039)	(0.039)	(0.075)	(0.050)	(0.056)	(0.091)
Higher education	0.177***	0.141***	0.267***	0.333***	0.259***	0.394***
	(0.041)	(0.045)	(0.076)	(0.053)	(0.063)	(0.089)
Children	− 0.011	− 0.022*	0.016	− 0.018	0.002	− 0.032
	(0.013)	(0.013)	(0.030)	(0.027)	(0.031)	(0.054)
Round 2	0.056**	0.030	0.094**	0.099***	0.069*	0.146**
	(0.024)	(0.029)	(0.039)	(0.037)	(0.041)	(0.067)
Round 3	0.213***	0.234***	0.189***	0.268***	0.258***	0.272***
	(0.032)	(0.045)	(0.043)	(0.042)	(0.053)	(0.064)
Constant	− 0.119***	− 0.133**	− 0.140*	− 0.113	− 0.132*	− 0.121
	(0.042)	(0.053)	(0.082)	(0.071)	(0.080)	(0.136)
Observations	3122	1573	1549	1857	949	908
R-squared	0.186	0.193	0.232	0.166	0.173	0.168

Coefficients are from linear models. Standard errors clustered at the regional level. Sampling weights applied. ***p < 0.01, **p < 0.05, *p < 0.1

**Table 3:** Determinants of knowledge of someone with COVID-19, Nigeria

Variables	(1)	(2)	(3)	(4)	(5)	(6)
	Women	Rural women	Urban women	Men	Rural men	Urban men
Migrated from 2018 to 2019 to Round 1 location	0.032	− 0.092**	0.107	0.006	0.022	− 0.001
	(0.050)	(0.043)	(0.076)	(0.028)	(0.046)	(0.032)
Single	− 0.025	− 0.052	− 0.002	− 0.092***	− 0.009	− 0.142***
	(0.021)	(0.034)	(0.024)	(0.031)	(0.042)	(0.041)
Widowed	− 0.000	− 0.043	0.021	− 0.167***	− 0.045	–
	(0.081)	(0.043)	(0.129)	(0.040)	(0.042)	
Separated–divorced	0.353*	0.084	0.397*	− 0.094*	− 0.037	0.120**
	(0.198)	(0.154)	(0.208)	(0.048)	(0.038)	(0.049)
Age 26–35	0.015	− 0.002	0.018	− 0.003	0.012	− 0.012
	(0.021)	(0.038)	(0.024)	(0.024)	(0.037)	(0.031)
Age 36–45	0.058	0.029	0.061	− 0.027	0.016	− 0.051
	(0.039)	(0.060)	(0.048)	(0.039)	(0.053)	(0.052)
Age 46–55	− 0.013	0.068	− 0.081**	− 0.023	− 0.040	− 0.028
	(0.048)	(0.077)	(0.035)	(0.050)	(0.061)	(0.067)
Age 56–65	0.129*	− 0.056	0.190*	− 0.084*	− 0.042	− 0.111*
	(0.070)	(0.035)	(0.102)	(0.047)	(0.062)	(0.063)
More than 65	0.355	0.060	0.461	0.402*	− 0.085*	0.482**
	(0.250)	(0.089)	(0.291)	(0.219)	(0.052)	(0.192)
Secondary education	− 0.021	0.031	− 0.079	0.083**	0.099***	0.072
	(0.037)	(0.037)	(0.059)	(0.037)	(0.031)	(0.072)
Higher education	0.076*	0.146**	0.019	0.139***	0.109***	0.142**
	(0.042)	(0.062)	(0.056)	(0.036)	(0.031)	(0.071)
Children	− 0.005	0.021*	− 0.017*	0.020**	0.022**	0.023*
	(0.008)	(0.012)	(0.010)	(0.008)	(0.010)	(0.013)
Round 2	0.016	− 0.007	0.020	− 0.017	− 0.017	− 0.015
	(0.013)	(0.018)	(0.017)	(0.018)	(0.027)	(0.025)
Round 3	0.049***	0.016	0.059**	0.061***	0.029	0.083***
	(0.018)	(0.025)	(0.024)	(0.021)	(0.032)	(0.029)
Constant	0.024	0.003	0.058	0.035	− 0.028	0.075
	(0.039)	(0.044)	(0.059)	(0.052)	(0.053)	(0.089)
Observations	2704	944	1760	2070	762	1308
R-squared	0.090	0.086	0.145	0.083	0.047	0.124

Coefficients are from linear models. Standard errors clustered at the regional level. Sampling weights applied. ***p < 0.01, **p < 0.05, *p < 0.1

Table 4 presents the results from model (2), which shows the association between lagged exposure to COVID at the regional level and current migration by gender of the respondent and destination. In Kenya, the transmission of COVID and policies targeting outbreaks might have hampered the ability of men and women to migrate for work. The point estimate on the COVID case variable is − 0.008 for men and − 0.009 for women. Comparing the point estimates relative to the mean migration rates (Table A8), the results indicate that migration rates declined by 3 and 5 percentage points for men and women with each additional case per 10,000 people in the region. The results are stronger for men and women moving across counties.

**Table 4:** Migration and COVID exposure relationship

	(1)	(2)	(3)	(4)	(5)	(6)	(7)	(8)	(9)
Outcome, sample	Migrate, All	Migrate, Men	Migrate, Women	Migrate within region, All	Migrate across regions, All	Migrate within region, Men	Migrate across regions, Men	Migrate within region, Women	Migrate across regions, Women
*Panel A: Kenya*									
Cases per 10,000 people	− 0.009***	− 0.008*	− 0.009***	0.000	− 0.009***	0.000	− 0.008**	0.000	− 0.010***
	(0.002)	(0.004)	(0.002)	(0.000)	(0.002)	(0.000)	(0.004)	(0.001)	(0.002)
Constant	0.089**	0.086	− 0.040	− 0.013**	0.102***	− 0.009	0.095	− 0.014	− 0.026
	(0.035)	(0.060)	(0.029)	(0.006)	(0.036)	(0.007)	(0.058)	(0.011)	(0.028)
Observations	2977	1127	1850	2977	2977	1127	1127	1850	1850
R-squared	0.693	0.678	0.703	0.571	0.715	0.599	0.687	0.562	0.733
*Panel B: Nigeria*									
Cases per 10,000 people	− 0.000	0.001	− 0.002*	0.000	− 0.000	− 0.000	0.001	0.000	− 0.002**
	(0.001)	(0.002)	(0.001)	(0.000)	(0.001)	(0.001)	(0.001)	(0.000)	(0.001)
Constant	0.014	0.522***	0.079*	− 0.001	0.015	0.520***	0.002	− 0.003	0.082**
	(0.038)	(0.026)	(0.044)	(0.016)	(0.030)	(0.014)	(0.019)	(0.018)	(0.040)
Observations	2861	1263	1598	2861	2861	1263	1263	1598	1598
R-squared	0.632	0.615	0.647	0.593	0.646	0.569	0.642	0.608	0.655

Coefficients are from linear models. Standard errors clustered at the regional level. Sampling weights applied. ***p < 0.01, **p < 0.05, *p < 0.1

Immobility across states appears to persist in Nigeria. However, the effect is mainly experienced by women at a lower level of magnitude. For example, the point estimate is − 0.002 in the specification that focuses on migration across states in Nigeria (column 9, Table 4). Comparing the point estimate to the mean migration rate across states for women (Table A8), the results indicate that exposure to an additional COVID case per 10,000 people would result in a decline in interstate migration among women by 2 percentage points. Taken together, the results from Table 4 suggest women faced constraints on their interregional mobility corroborating our second hypothesis.

## Alternative Models and Robustness Checks

The previous specifications do not take advantage of the complete migration history collected in the survey to describe who might have experienced a greater transition during the pandemic in terms of their barriers to movement in the short term. The fixed effect specifications also absorb all time-invariant individual characteristics, limiting our ability to examine which demographic groups might have experienced greater restrictions on their movement as COVID-19 policies were implemented. We therefore provide estimates of a series of linear probability models using the migration outcomes (migrate, migrate within region, migrate to another region)^2^ and covariates collected at baseline, which include indicators for gender (female; male is the omitted category), marital status (single, widowed, separated/divorced; married is omitted category), age (26–35, 36–45, 46–55, 56–65,  > 65; 18–25 is omitted category), education (secondary, higher education; omitted category is less than secondary education), the number of children (pre-primary school and below) in the household, and year (2020, 2021; omitted year is 2019). We further include the variables that interact the baseline demographic characteristics and year to determine whether specific demographic groups experienced greater changes in magnitude over the pandemic years.^3^ The coefficients and statistical significance of the variables and their interaction terms in each model are presented in Table 5 (Table A9 includes the coefficients and their respective standard errors).

**Table 5:** Determinants of Migration in the Last 12 Months, Kenya and Nigeria with interactions

Variables	(1)	(2)	(3)	(4)	(5)	(6)
	Migrate in Kenya	Migrate within county in Kenya	Migrate to another county in Kenya	Migrate in Nigeria	Migrate within state in Nigeria	Migrate to another state in Nigeria
Female	− 0.023	0.000	− 0.024	0.013	0.001	0.019
Female × 2020	− 0.070	0.001	− 0.070	− 0.024	− 0.000	− 0.030
Female × 2021	− 0.035	− 0.017	− 0.023	− 0.025	− 0.001	− 0.031
Single	− 0.014	− 0.012	− 0.002	0.048	0.016	0.030
Single × 2020	0.020	− 0.001	0.019	0.031	0.029	0.004
Single × 2021	0.190***	0.039	0.157**	0.073	− 0.016	0.091**
Widowed	− 0.057	− 0.033*	− 0.024	− 0.028	− 0.015	− 0.015
Widowed × 2020	0.114	− 0.007	0.121	0.024	0.001	0.026
Widowed × 2021	0.009	− 0.030	0.042	− 0.061	0.015	− 0.074
Separated/Divorced	− 0.082	− 0.027	− 0.054	0.214*	0.070	0.147
Separated/Divorced × 2020	0.235*	0.170	0.065	− 0.067	− 0.074	0.005
Separated/Divorced × 2021	0.034	0.048	− 0.014	− 0.154	− 0.070	− 0.087
Age 26–35	− 0.039	0.007	− 0.047	0.030	0.046*	− 0.010
Age 26–35 × 2020	− 0.075	− 0.016	− 0.061	− 0.060**	− 0.037**	− 0.029
Age 26–35 × 2021	− 0.108	− 0.032	− 0.080	− 0.074	− 0.046*	− 0.034
Age 36–45	− 0.190***	− 0.033**	− 0.159***	− 0.065	− 0.006	− 0.056**
Age 36–45 × 2020	− 0.062	0.015	− 0.078	0.007	0.019	− 0.015
Age 36–45 × 2021	0.129	0.053	0.070	− 0.018	0.006	− 0.027
Age 46–55	− 0.097	0.051	− 0.148**	− 0.081	0.024	− 0.099***
Age 46–55 × 2020	− 0.131*	− 0.016	− 0.116	− 0.004	− 0.011	0.002
Age 46–55 × 2021	− 0.099	− 0.050	− 0.055	− 0.037	− 0.024	− 0.019
Age 56–65	− 0.227***	− 0.025	− 0.202***	− 0.066	− 0.023	− 0.035
Age 56–65 × 2020	− 0.112	− 0.021	− 0.092	0.027	0.036	− 0.017
Age 56–65 × 2021	0.040	− 0.007	0.041	0.096	0.023	0.065
More than 65	0.065	− 0.031	0.095	− 0.167***	− 0.062**	− 0.103***
More than 65 × 2020	− 0.421*	0.010	− 0.432*	0.042	0.022	0.018
More than 65 × 2021	− 0.286	− 0.023	− 0.268	0.061	0.062**	− 0.003
Secondary education	0.016	− 0.006	0.021	− 0.066	− 0.030	− 0.045
Secondary education × 2020	0.067	0.035	0.031	0.102***	0.039	0.072**
Secondary education × 2021	− 0.035	− 0.035	0.003	0.159***	0.030	0.138**
Higher education	− 0.002	0.002	− 0.004	0.033	0.021	0.012
Higher education × 2020	0.187***	0.064*	0.123**	0.023	− 0.003	0.025
Higher education × 2021	0.102	0.023	0.076	0.047	− 0.021	0.068
Children	− 0.004	− 0.011*	0.008	− 0.010	− 0.002	− 0.005
Children × 2020	− 0.006	0.006	− 0.012	0.003	0.003	− 0.001
Children × 2021	0.038	0.006	0.030	0.009	0.002	0.004
2020	0.061	− 0.031	0.092	− 0.060	− 0.029	− 0.023
2021	0.016	0.033	− 0.008	− 0.059	− 0.056	0.005
Constant	0.263***	0.052*	0.212**	0.165***	0.056	0.101**
Observations	5333	5333	5333	5105	5105	5105
R-squared	0.097	0.028	0.086	0.042	0.034	0.064

Coefficients are from linear models.Standard errors clustered at the regional level. ***p < 0.01, **p < 0.05, *p < 0.1

The results in Table 5 suggest that the capacity to move during the pandemic changed with marital status, education, and age. A resounding finding across countries is that individuals who were single were more inclined to move in 2021, especially when restricting the focus to migrating to another county or state. The coefficients in Table 5 suggest that the probability of moving to another region if one is single increased 0.16 percentage points in Kenya and 0.09 percentage points in Nigeria relative to individuals with other marital statuses in 2021. Mobility also was higher among those with a secondary education (in Nigeria) and tertiary education (in Kenya) relative to those in absence of such an education in 2020 and 2021. In contrast, those most vulnerable to the pandemic in terms of facing constraints on mobility were youth (26–35 years old) in Nigeria and mature adults (46–55 years old) in Kenya.

We also extend the results in Table 3 to consider how the type of migration may influence the estimated relationship between awareness of someone that has COVID-19 in one’s network and pre-pandemic migration. The expected signs of moving within region or across region are ambiguous. They depend on the relative importance of specific factors that determine migrant wealth and the potential for the migrant to act on any additional health knowledge gained from their previous location. We might expect the distance of migration to be associated with reducing variability in income and hence raising the expected wealth to avert risk (Dillon et al., 2011). Yet, the costs required to move across regions and cost of living expenses (in urban locations) can also serve to lower the expected return from migration (Sjaastad, 1962; Massey et al., 1993). We might therefore anticipate the relationship to be dampened when directing the focusing to interregional migration or residents in urban areas.

There is limited evidence to support the information mechanism particularly in the context of internal migration. We expect this channel to be strongest when studying discrepancies between international migrants and non-migrants, given disparities in health knowledge between origins and destinations abroad. However, Valsecchi and Durante (2021) establish a precedence for investigating this channel in the context of internal migration. They highlight that urban areas in the South of Italy observed strong negative effects on mobility during the pandemic because the region already had low local mobility and, thus, social distancing may have achieved the highest impact given its population density.

Our findings are consistent with the presence of both wealth and information channels in Kenya and Nigeria (Tables A10 and A11). In Kenya, the results indicate that male migrants living in urban locations who moved within the county prior to Round 1 are 15 percentage points less likely to know of someone in their network with the disease. In Nigeria, rural women who migrated within the same state were 13 percentage points less likely to know someone with COVID-19. Both case studies provide support for our first hypothesis when differentiating the effects by the type of move. The findings suggest that the wealth effects and/or knowledge acquired through migrant networks for urban men in Kenya and rural women in Nigeria may mediate any potential systematic risks associated with moving across locations.

## Data Limitations

Although our research offers some of the first insights on mobility and vulnerability to COVID-19 in Africa, it faces a few limitations. First, one potential drawback in using the RDD approach is that disparities in mobile phone ownership can skew the composition of the sample (Glazerman et al., 2023). Mueller et al. (2022) and Glazerman et al. (2023) illustrate that the RDD samples used here obtain greater levels of education and higher tendencies for employment relative to a representative population. Survey sampling weights were designed and applied to partially adjust for these discrepancies using the method in Kastelic et al. (2020). However, the authors note that the survey weights may not entirely correct for the distortions in the data. Thus, our results are unlikely generalizable to the broader population, particularly to groups that are asset poor, and the selection bias may be stronger for women relative to men given discrepancies in phone ownership in Kenya and Nigeria and the differential attrition rates noted in the Data section.

Second, the outcome in model 1 may be subject to measurement error as it captures perceived risk in one’s network rather than objective risk in one’s network. Mueller et al. (2022) show that the individual measure of perceived risk is positively correlated with an objective measure of regional-level COVID cases in Kenya. One possibility is that the perceived risk outcome in Nigeria is subject to skewed measurement error. For example, those who declare knowing someone with COVID-19 may disproportionately over report risk relative to those who misreport not knowing someone with COVID-19, or vice versa. While classical measurement results in a loss of precision in the estimates, ignoring skewed measurement error in the outcome can lead to biased estimates which are particularly pronounced when heteroskedasticity is extant (Millimet & Parmeter, 2022). Since we are unable to match the individual risk measure with actual risk at a level finer than the county/state, we are unable to gauge the severity of individual measurement error (and whether it is skewed). We therefore recognize this limitation in our analysis. Future studies that have access to subjective and objective measures of risk over the same unit of analysis may consider applying the recommendations of Millimet and Parmeter (2022), which are to use stochastic frontier analysis or nonlinear least squares models to solve the problem.

Third, our analysis is mainly descriptive given the nature of data collection over mobile phones at the time of the pandemic. We were quite cognizant of the need to maintain short interviews, which limited the number of questions that could be asked and answered by the respondent. In model 2, for example, the absence of important control variables (e.g., migrant networks, exposure to idiosyncratic shocks) is exacerbated by the low temporal variation in those variables that are collected (e.g., education, household size) due to the short duration of the study. We emphasize here, and, throughout the text, that our relationships are clearly reflecting associations given the potential for omitted variable bias. In an individual fixed effect model, one source of omitted variable bias might arise from the exclusion of other time-variant shocks that may be correlated with the rate of COVID-19 at the regional level. Presumably places with higher lagged incidence of COVID-19 might also disproportionately be more vulnerable to income shocks due to the lack of medical infrastructure (affecting labor productivity) or the concentration of service-oriented industries in the vicinity (affecting labor demand). Excluding controls for these regional-level factors might induce a negative bias on the risk coefficient in model 2. Thus, the negative estimates displayed in Table 4 may, in fact, be overestimating the negative relationship estimated between migration and lagged region-level COVID rates. Future work can alleviate concerns over omitted variable bias by using an instrumental variable approach, where the candidate instrumental variable can be derived from basic epidemiological models. Once local measures of the infection rate, contact rate, and cases become available to the public, such an instrument can be constructed in extensions of this work.

## Discussion and Conclusions

Migrants moved to the forefront of humanitarian concerns during the pandemic. The literature on the pandemic suggests that migrants typically face greater health risks due to differences in exposure and wealth at the destination (Lienhardt, 2001; Mari et al., 2012). Although the same vulnerabilities reoccurred amidst the COVID-19 pandemic (Cruz et al., 2020; Gupta et al., 2021; Khan & Arokkiaraj, 2021), there was a lack of evidence from Africa at the time of our investigation. Furthermore, most studies ignored the differential health threat incurred by migrant women relative to non-migrant women and their male counterparts. Arguably, by virtue of the type of work that women do in the informal sector, they may face greater threats on their health. Many migrant women are employed in the service sector, where their earnings depend on providing goods and services to customers in person rather than remotely. Others work in the manufacturing industry and perform tasks in congested quarters. The working conditions in these female-dominated sectors alone suggest that migrant women may be at greater risk of contracting COVID-19 than other populations.

We utilized longitudinal data collected in Kenya and Nigeria to first understand whether the health risks of migrant women were more vulnerable than other demographic groups. We proxied their own health risk by whether they were more likely to know individuals in their network who contracted the disease. We find the opposite of what the pandemic literature would predict. There is a negative association between internal migration and knowledge of someone with COVID-19 in your own network. These findings may be attributed to the mechanisms discussed in the migration literature. Although migrants may have greater wealth to afford strategies that avert risk (Beegle et al., 2011; Hirvonen, 2016; Lagakos et al., 2020), evidence of the altruistic tendencies of migrants (De La Brière et al., 2002; Vanwey, 2004) and their ability to acquire health knowledge from their networks (Hildebrandt & McKenzie, 2005) has largely been supported for women rather than men. Women migrants may be more inclined to use their additional wealth to avert risk and reduce communal risk.

We next analyzed the longitudinal data to examine the extent to which the mobility of women might have been tempered by the pandemic. We find that interregional migration was hindered during the pandemic, especially for women in Kenya and Nigeria. Exposure to an additional COVID case per 10,000 people resulted in a decline in women’s interregional migration by 6 and 2 percentage points relative to the mean migration rates in Kenya and Nigeria, respectively. Although national school closures were prevalent in both contexts, they had ended by the second and third rounds of our study. During the later rounds of our survey, curfews were in effect making it harder to travel at certain times of day. Thus, there were two phenomena potentially underlying the observed migration relationships. First, would-be migrant women with young children living in the household likely substituted paid for unpaid work during national school closures. Second, additional restrictions on the timing of movement affected women’s ability to migrate for work directly and indirectly. Women were directly affected as the curfews reduced the flexibility of their schedules, perhaps determining whether they could remain in the labor force. Women were indirectly affected as the curfews might have made it more difficult to outsource the increasing burden of unpaid work by relying on older family members or hiring caregivers (with similar constraints) (Camilletti & Nesbitt-Ahmed, 2022).

A natural question becomes how governments might protect women who rely on migration for employment. This requires understanding through which pathways governments can mitigate the potential costs of social distancing policies, which include migrant women’s loss of income through exiting the labor force or adjusting their work hours as well as interruptions in their work experience and skill acquisition which alters their stream of income. Certain social protection schemes, such as unemployment insurance, are often not accessible to workers employed in the informal sector. Hidrobo et al. (2020) suggest expanding the targeted beneficiaries of these type of programs to protect workers in the informal sector, which largely consist of women. Offering cash injections will resolve short-term constraints on liquidity yet fails to address the constraints affecting women’s ability to work in the long term. Camilletti and Nesbitt-Ahmed (2022) argue that governments should increase the provision of quality childcare to reduce the demand for unpaid work, as well as encourage employers to provide more flexible work arrangements. Furthermore, they should also create benefits to attract workers into paid care and domestic services, such as ensuring a living wage, subsidizing childcare for those in this industry, and offering paid family and sick leave. Such policies can both reduce the effects of the pandemic and foster the economic growth of low-income countries through the increased labor productivity provided by women.

## Data Availability

The longitudinal survey data collected in Kenya and Nigeria are publically available on the Harvard Dataverse website at: https://doi.org/10.7910/DVN/ODM6I0.

## Appendix

See Tables

**Table A1:** Decomposition of attrition by round and country

	Observations	w/non-missing data
*Kenya*		
Round 1 only	355	355
Rounds 1 and 2 only	316	316
Rounds 1, 2, 3	1331	1331
Total sample	2002	2002
*Nigeria*		
Round 1 only	324	322
Rounds 1 and 2 only	365	362
Rounds 1, 2, 3	1248	1246
Total sample	1937	1930

The non-missing data column includes observations which have information pertaining to the respondent’s gender, marital status, age, education level, number of children, wealth score, and current location

A1,

**Table A2:** Attrition analysis for Kenya and Nigeria

Variables	(1)	(2)	(3)	(4)
	Attrition for Round 1 and 2 in Nigeria	Attrition for Round 1, 2 and 3 in Nigeria	Attrition for Round 1 and 2 in Kenya	Attrition for Round 1, 2 and 3 in Kenya
Female	0.0381	0.0860***	− 0.0405	0.0306
	(0.0461)	(0.0251)	(0.0716)	(0.0354)
Single	− 0.0127	0.00985	− 0.0986	0.0622
	(0.0563)	(0.0322)	(0.0920)	(0.0438)
Separated–Divorced	0.164	− 0.0432	− 0.218	− 0.0651
	(0.121)	(0.120)	(0.191)	(0.0442)
Widowed	− 0.816***	− 0.276***	0.334**	0.0526
	(0.153)	(0.0455)	(0.143)	(0.117)
Age 26–35	0.122**	0.0952***	− 0.161*	− 0.217***
	(0.0548)	(0.0264)	(0.0888)	(0.0404)
Age 36–45	0.149*	0.105**	− 0.0468	− 0.208***
	(0.0853)	(0.0423)	(0.139)	(0.0520)
Age 46–55	0.575***	0.261***	0.183	− 0.0266
	(0.0742)	(0.0770)	(0.124)	(0.0793)
Age 56–65	0.0405	0.00514	− 0.190	− 0.142
	(0.203)	(0.0955)	(0.196)	(0.0893)
More than 65	0.735***	0.140	− 0.358	0.462**
	(0.141)	(0.192)	(0.254)	(0.231)
Primary Education	0.154	− 0.0640	− 0.0405	0.0995
	(0.130)	(0.117)	(0.219)	(0.124)
Secondary Education	0.103	− 0.160*	− 0.128	0.0156
	(0.101)	(0.0932)	(0.223)	(0.118)
Higher Education	0.0777	− 0.270***	− 0.157	− 0.0431
	(0.102)	(0.0925)	(0.229)	(0.119)
Children	0.00616	0.00413	− 0.0482**	0.0299
	(0.0214)	(0.00869)	(0.0201)	(0.0256)
Wealth Score Quintile 2	0.0719	0.0216	− 0.00335	0.00240
	(0.0691)	(0.0340)	(0.112)	(0.0559)
Wealth Score Quintile 3	0.0640	0.0338	0.0404	0.0374
	(0.0785)	(0.0377)	(0.115)	(0.0530)
Wealth Score Quintile 4	− 0.00976	0.0395	− 0.0732	0.0708
	(0.0668)	(0.0380)	(0.109)	(0.0623)
Wealth Score Quintile 5	0.0563	0.107***	0.0147	0.0383
	(0.0723)	(0.0411)	(0.111)	(0.0599)
Rural	0.0189	− 0.0328	− 0.0411	0.0457
	(0.0472)	(0.0246)	(0.0699)	(0.0372)
Constant	0.184	0.263***	0.850***	0.185
	(0.130)	(0.0963)	(0.236)	(0.125)
Observations	684	1568	671	1686
R-squared	0.125	0.081	0.078	0.098

Columns 1 and 3 evaluate attrition from round 1 to round 2. The dependent variable assigns a zero to people that were interviewed in rounds 1 and 2, and one to people that were only interviewed in round 1. Columns 2 and 4 evaluate attrition from rounds 1 to 3. The dependent variable assigns a zero to people that were interviewed in rounds 1, 2, and 3, and one to people that were only interviewed in round 1. Sampling weights included. Coefficients are from linear models. Standard errors clustered by region in parentheses. ****p* < 0.01, ***p* < 0.05, **p* < 0.1

A2,

**Table A3:** Sample composition for analysis of determinants of migration, including pre-COVID year

	Observations	Total panel obs	w/ non-missing data
*Kenya*			
Round 1 only	355	710	709
Rounds 1 and 2 only	316	632	631
Rounds 1, 2, 3	1331	3993	3993
Total sample	2002	5335	5333
*Nigeria*			
Round 1 only	324	648	644
Rounds 1 and 2 only	365	730	724
Rounds 1, 2, 3	1248	3744	3737
Total sample	1937	5122	5105

The non-missing data column includes observations which have information pertaining to the respondent’s gender, marital status, age, education level, number of children, wealth score, and current location

A3,

**Table A4:** Sample composition for determinants of knowledge of someone with COVID-19 analysis

	Observations	Total panel obs	w/ non-missing data
*Kenya*			
Round 1 only	355	355	354
Rounds 1 and 2 only	316	632	632
Rounds 1, 2, 3	1331	3993	3993
Total sample	2002	4980	4979
*Nigeria*			
Round 1 only	324	324	321
Rounds 1 and 2 only	365	730	724
Rounds 1, 2, 3	1248	3744	3729
Total sample	1937	4798	4774

The non-missing data column includes observations which have information pertaining to the respondent’s gender, marital status, age, education level, number of children, wealth score, current location, migration history, and knowledge of someone with COVID-19

A4,

**Table A5:** Summary statistics, Kenya

	Kenya						
	Total	Women	Rural women	Urban women	Men	Rural men	Urban men
Migrated from 2018 to 2019 to Round 1 location	0.28	0.28	0.30	0.24	0.29	0.30	0.28
Migrated within county from 2018 to 2019 to Round 1 location	0.04	0.04	0.04	0.03	0.04	0.05	0.03
Migrated to another county from 2018 to 2019 to Round 1 location	0.24	0.24	0.26	0.21	0.25	0.25	0.25
Female	0.64	1.00	1.00	1.00	0.00	0.00	0.00
Married	0.38	0.41	0.42	0.39	0.35	0.32	0.40
Single	0.55	0.51	0.53	0.50	0.62	0.66	0.57
Widowed	0.03	0.04	0.04	0.04	0.02	0.01	0.02
Divorce or separated	0.03	0.04	0.01	0.07	0.01	0.02	0.01
Age 15–25	0.64	0.63	0.65	0.59	0.66	0.70	0.61
Age 26–35	0.16	0.16	0.12	0.22	0.15	0.15	0.17
Age 36–45	0.08	0.08	0.08	0.07	0.09	0.08	0.11
Age 46–55	0.07	0.08	0.09	0.07	0.06	0.05	0.08
Age 56–65	0.04	0.04	0.04	0.04	0.03	0.03	0.03
Age more than 65	0.01	0.01	0.01	0.00	0.01	0.00	0.01
Not formal education	0.00	0.00	0.00	0.00	0.00	0.00	0.00
Pre-Primary education	0.01	0.00	0.00	0.01	0.01	0.01	0.00
Primary education	0.12	0.14	0.15	0.12	0.11	0.10	0.12
Secondary education	0.45	0.44	0.44	0.43	0.47	0.52	0.39
Higher education	0.42	0.42	0.41	0.44	0.42	0.37	0.50
Baseline: number of young children	0.78	0.89	0.97	0.78	0.59	0.64	0.51
Wealth Score in Round 1	− 0.56	− 0.50	− 0.84	− 0.00	− 0.67	− 1.17	0.07
Wealth quintile 1	0.29	0.26	0.31	0.19	0.34	0.43	0.22
Wealth quintile 2	0.22	0.25	0.27	0.22	0.17	0.18	0.16
Wealth quintile 3	0.20	0.19	0.20	0.18	0.20	0.21	0.20
Wealth quintile 4	0.17	0.19	0.17	0.21	0.15	0.11	0.21
Wealth quintile 5	0.12	0.11	0.05	0.20	0.14	0.08	0.21
Know someone infected with COVID-19	0.27	0.26	0.23	0.30	0.30	0.24	0.38
Rural	0.59	0.59	1.00	0.00	0.60	1.00	0.00
Round 1	0.35	0.35	0.38	0.31	0.35	0.39	0.29
Round 2	0.34	0.34	0.34	0.35	0.33	0.33	0.34
Round 3	0.31	0.30	0.28	0.33	0.32	0.29	0.37
*N*	4979	3122	1573	1549	1857	949	908

All explanatory variables are defined from the baseline survey except the year and rural indicators. Sampling weights applied

A5,

**Table A6:** Summary statistics, Nigeria

	Nigeria						
	Total	Women	Rural women	Urban women	Men	Rural men	Urban men
Migrated from 2018 to 2019 to Round 1 location	0.13	0.14	0.15	0.13	0.13	0.13	0.13
Migrated within state from 2018 to 2019 to Round 1 location	0.07	0.07	0.09	0.06	0.07	0.06	0.07
Migrated to another state from 2018 to 2019 to Round 1 location	0.06	0.07	0.07	0.07	0.06	0.07	0.06
Female	0.52	1.00	1.00	1.00	0.00	0.00	0.00
Married	0.60	0.59	0.60	0.59	0.60	0.61	0.59
Single	0.36	0.32	0.33	0.32	0.39	0.36	0.41
Widowed	0.03	0.06	0.06	0.06	0.00	0.01	0.00
Divorce or separated	0.01	0.02	0.01	0.02	0.01	0.02	0.00
Age 15–25	0.23	0.23	0.24	0.23	0.23	0.24	0.22
Age 26–35	0.29	0.31	0.30	0.31	0.27	0.26	0.27
Age 36–45	0.20	0.22	0.18	0.25	0.18	0.20	0.17
Age 46–55	0.17	0.14	0.21	0.10	0.20	0.19	0.21
Age 56–65	0.09	0.08	0.06	0.10	0.10	0.10	0.10
Age more than 65	0.02	0.02	0.01	0.02	0.01	0.01	0.02
Not formal education	0.03	0.03	0.05	0.02	0.04	0.05	0.03
Pre-Primary education	0.00	0.00	0.00	0.00	0.00	0.01	0.00
Primary education	0.06	0.07	0.07	0.07	0.05	0.07	0.03
Secondary education	0.32	0.33	0.40	0.29	0.32	0.42	0.26
Higher education	0.58	0.57	0.48	0.62	0.60	0.45	0.68
Baseline: number of young children	0.90	0.73	0.91	0.63	1.09	1.47	0.87
Wealth Score in Round 1	− 0.02	− 0.08	− 0.41	0.10	0.04	− 0.58	0.40
Wealth quintile 1	0.22	0.23	0.30	0.19	0.21	0.30	0.15
Wealth quintile 2	0.18	0.17	0.22	0.15	0.18	0.21	0.17
Wealth quintile 3	0.20	0.20	0.10	0.25	0.19	0.20	0.19
Wealth quintile 4	0.19	0.20	0.24	0.17	0.18	0.16	0.19
Wealth quintile 5	0.22	0.20	0.14	0.23	0.24	0.12	0.30
Know someone infected with COVID-19	0.12	0.11	0.09	0.13	0.13	0.09	0.15
Rural	0.37	0.36	1.00	0.00	0.37	1.00	0.00
Round 1	0.33	0.33	0.34	0.33	0.33	0.33	0.33
Round 2	0.34	0.34	0.33	0.34	0.34	0.33	0.34
Round 3	0.33	0.33	0.33	0.33	0.33	0.33	0.33
*N*	4774	2704	944	1760	2070	762	1308

All explanatory variables are defined from the baseline survey except the year and rural indicators. Sampling weights applied

A6,

**Table A7:** Sample composition for quantifying COVID-19 exposure and migration relationship

	Observations	Total panel obs	w/non-missing data
*Kenya*			
Round 1 only	355	0	0
Rounds 1 and 2 only	315	315	315
Rounds 1, 2, 3	1331	2662	2662
Total sample	2002	2978	2977
*Nigeria*			
Round 1 only	324	0	0
Rounds 1 and 2 only	365	365	362
Rounds 1, 2, 3	1248	2496	2496
Total sample	1969	2861	2861

The non-missing data column includes observations which have information pertaining to the respondent’s gender, marital status, age, education level, number of children, wealth score, current location, migration history, and knowledge of someone with COVID-19

A7,

**Table A8:** Summary statistics

	Kenya			Nigeria		
	Total	Women	Men	Total	Women	Men
Migrated since previous round	0.21	0.20	0.23	0.12	0.13	0.12
Migrated within region since previous round	0.02	0.03	0.01	0.03	0.03	0.03
Migrated to another region since previous round	0.19	0.17	0.22	0.09	0.09	0.09
Female	0.63	1.00	0.00	0.58	1.00	0.00
Cumulative new cases per 10,000 people since last round (Covid-19)	15.61	15.26	16.23	12.13	12.10	12.17
Round 2	0.52	0.53	0.51	0.51	0.51	0.51
Round 3	0.48	0.47	0.49	0.49	0.49	0.49
*N*	2977	1850	1127	2861	1598	1263

Sampling weights applied

A8,

**Table A9:** Table 2, with standard errors

Variables	(1)	(2)	(3)	(4)	(5)	(6)
	Migrate in Kenya	Migrate within county in Kenya	Migrate to another county in Kenya	Migrate in Nigeria	Migrate within state in Nigeria	Migrate to another state in Nigeria
Female	− 0.023	0.000	− 0.024	0.013	0.001	0.019
	(0.041)	(0.017)	(0.039)	(0.026)	(0.019)	(0.018)
Female × 2020	− 0.070	0.001	− 0.070	− 0.024	− 0.000	− 0.030
	(0.048)	(0.018)	(0.046)	(0.022)	(0.015)	(0.019)
Female × 2021	− 0.035	− 0.017	− 0.023	− 0.025	− 0.001	− 0.031
	(0.067)	(0.034)	(0.061)	(0.038)	(0.019)	(0.037)
Single	− 0.014	− 0.012	− 0.002	0.048	0.016	0.030
	(0.051)	(0.017)	(0.050)	(0.034)	(0.029)	(0.022)
Single × 2020	0.020	− 0.001	0.019	0.031	0.029	0.004
	(0.061)	(0.029)	(0.056)	(0.029)	(0.020)	(0.023)
Single × 2021	0.190***	0.039	0.157**	0.073	− 0.016	0.091**
	(0.071)	(0.029)	(0.068)	(0.046)	(0.029)	(0.042)
Widowed	− 0.057	− 0.033*	− 0.024	− 0.028	− 0.015	− 0.015
	(0.054)	(0.020)	(0.052)	(0.060)	(0.047)	(0.035)
Widowed × 2020	0.114	− 0.007	0.121	0.024	0.001	0.026
	(0.144)	(0.012)	(0.146)	(0.023)	(0.019)	(0.017)
Widowed × 2021	0.009	− 0.030	0.042	− 0.061	0.015	− 0.074
	(0.102)	(0.031)	(0.105)	(0.082)	(0.047)	(0.070)
Separated/Divorced	− 0.082	− 0.027	− 0.054	0.214*	0.070	0.147
	(0.056)	(0.020)	(0.052)	(0.120)	(0.085)	(0.095)
Separated/Divorced × 2020	0.235*	0.170	0.065	− 0.067	− 0.074	0.005
	(0.134)	(0.138)	(0.078)	(0.071)	(0.071)	(0.016)
Separated/Divorced × 2021	0.034	0.048	− 0.014	− 0.154	− 0.070	− 0.087
	(0.101)	(0.075)	(0.063)	(0.135)	(0.085)	(0.094)
Age 26–35	− 0.039	0.007	− 0.047	0.030	0.046*	− 0.010
	(0.054)	(0.017)	(0.052)	(0.033)	(0.024)	(0.024)
Age 26–35 × 2020	− 0.075	− 0.016	− 0.061	− 0.060**	− 0.037**	− 0.029
	(0.070)	(0.030)	(0.065)	(0.030)	(0.017)	(0.027)
Age 26–35 × 2021	− 0.108	− 0.032	− 0.080	− 0.074	− 0.046*	− 0.034
	(0.073)	(0.031)	(0.070)	(0.046)	(0.024)	(0.047)
Age 36–45	− 0.190***	− 0.033**	− 0.159***	− 0.065	− 0.006	− 0.056**
	(0.057)	(0.016)	(0.057)	(0.041)	(0.035)	(0.027)
Age 36–45 × 2020	− 0.062	0.015	− 0.078	0.007	0.019	− 0.015
	(0.064)	(0.035)	(0.056)	(0.030)	(0.019)	(0.025)
Age 36–45 × 2021	0.129	0.053	0.070	− 0.018	0.006	− 0.027
	(0.094)	(0.044)	(0.085)	(0.058)	(0.035)	(0.048)
Age 46–55	− 0.097	0.051	− 0.148**	− 0.081	0.024	− 0.099***
	(0.091)	(0.076)	(0.060)	(0.055)	(0.049)	(0.027)
Age 46–55 × 2020	− 0.131*	− 0.016	− 0.116	− 0.004	− 0.011	0.002
	(0.076)	(0.030)	(0.071)	(0.042)	(0.036)	(0.025)
Age 46–55 × 2021	− 0.099	− 0.050	− 0.055	− 0.037	− 0.024	− 0.019
	(0.129)	(0.100)	(0.089)	(0.071)	(0.049)	(0.055)
Age 56–65	− 0.227***	− 0.025	− 0.202***	− 0.066	− 0.023	− 0.035
	(0.056)	(0.019)	(0.055)	(0.058)	(0.037)	(0.045)
Age 56–65 × 2020	− 0.112	− 0.021	− 0.092	0.027	0.036	− 0.017
	(0.070)	(0.029)	(0.064)	(0.033)	(0.031)	(0.039)
Age 56–65 × 2021	0.040	− 0.007	0.041	0.096	0.023	0.065
	(0.090)	(0.031)	(0.087)	(0.100)	(0.037)	(0.099)
More than 65	0.065	− 0.031	0.095	− 0.167***	− 0.062**	− 0.103***
	(0.242)	(0.020)	(0.245)	(0.040)	(0.026)	(0.029)
More than 65 × 2020	− 0.421*	0.010	− 0.432*	0.042	0.022	0.018
	(0.229)	(0.030)	(0.233)	(0.032)	(0.018)	(0.028)
More than 65 × 2021	− 0.286	− 0.023	− 0.268	0.061	0.062**	− 0.003
	(0.254)	(0.039)	(0.254)	(0.090)	(0.026)	(0.087)
Secondary education	0.016	− 0.006	0.021	− 0.066	− 0.030	− 0.045
	(0.059)	(0.024)	(0.057)	(0.045)	(0.033)	(0.033)
Secondary education × 2020	0.067	0.035	0.031	0.102***	0.039	0.072**
	(0.052)	(0.028)	(0.045)	(0.038)	(0.025)	(0.032)
Secondary education × 2021	− 0.035	− 0.035	0.003	0.159***	0.030	0.138**
	(0.093)	(0.039)	(0.085)	(0.059)	(0.033)	(0.057)
Higher education	− 0.002	0.002	− 0.004	0.033	0.021	0.012
	(0.058)	(0.027)	(0.054)	(0.045)	(0.034)	(0.031)
Higher education × 2020	0.187***	0.064*	0.123**	0.023	− 0.003	0.025
	(0.058)	(0.034)	(0.049)	(0.037)	(0.026)	(0.028)
Higher education × 2021	0.102	0.023	0.076	0.047	− 0.021	0.068
	(0.093)	(0.044)	(0.084)	(0.058)	(0.034)	(0.053)
Children	− 0.004	− 0.011*	0.008	− 0.010	− 0.002	− 0.005
	(0.018)	(0.007)	(0.018)	(0.010)	(0.008)	(0.006)
Children × 2020	− 0.006	0.006	− 0.012	0.003	0.003	− 0.001
	(0.021)	(0.008)	(0.020)	(0.007)	(0.005)	(0.006)
Children × 2021	0.038	0.006	0.030	0.009	0.002	0.004
	(0.032)	(0.015)	(0.030)	(0.011)	(0.008)	(0.014)
2020	0.061	− 0.031	0.092	− 0.060	− 0.029	− 0.023
	(0.081)	(0.042)	(0.071)	(0.050)	(0.031)	(0.043)
2021	0.016	0.033	− 0.008	− 0.059	− 0.056	0.005
	(0.132)	(0.050)	(0.123)	(0.073)	(0.043)	(0.071)
Constant	0.263***	0.052*	0.212**	0.165***	0.056	0.101**
	(0.090)	(0.028)	(0.090)	(0.060)	(0.043)	(0.045)
Observations	5333	5333	5333	5105	5105	5105
R-squared	0.097	0.028	0.086	0.042	0.034	0.064

Coefficients are from linear models. Standard errors clustered at the regional level. Sampling weights applied. ****p* < 0.01, ***p* < 0.05, **p* < 0.1

A9,

**Table A10:** Determinants of knowledge of someone with COVID-19, Kenya

Variables	(1)	(2)	(3)	(4)	(5)	(6)
	Women	Rural women	Urban women	Men	Rural men	Urban men
Migrated within county from 2018 to 2019 to Round 1 location	− 0.003	− 0.002	0.061	0.012	0.106	− 0.153*
	(0.065)	(0.068)	(0.132)	(0.104)	(0.133)	(0.092)
Migrated to another county from 2018 to 2019 to Round 1 location	0.054	0.071	0.030	0.010	0.082	− 0.078
	(0.048)	(0.060)	(0.065)	(0.047)	(0.054)	(0.080)
Single	0.089***	0.136***	0.024	0.012	0.042	0.012
	(0.032)	(0.036)	(0.055)	(0.057)	(0.064)	(0.114)
Widowed	0.079	− 0.098	0.256**	− 0.521***	− 0.336***	− 0.704***
	(0.102)	(0.081)	(0.112)	(0.153)	(0.103)	(0.216)
Separated–Divorced	− 0.057	− 0.090	− 0.027	0.133	0.263	0.093
	(0.108)	(0.116)	(0.143)	(0.136)	(0.181)	(0.144)
Age 26–35	0.162***	0.170***	0.135*	0.153**	0.171**	0.168*
	(0.041)	(0.037)	(0.069)	(0.063)	(0.067)	(0.102)
Age 36–45	0.387***	0.355***	0.430***	0.332***	0.270***	0.400***
	(0.053)	(0.049)	(0.090)	(0.093)	(0.084)	(0.150)
Age 46–55	0.384***	0.460***	0.364***	0.255***	0.172**	0.348**
	(0.057)	(0.061)	(0.093)	(0.084)	(0.086)	(0.138)
Age 56–65	0.594***	0.471***	0.716***	0.319**	0.429***	0.209
	(0.080)	(0.100)	(0.068)	(0.132)	(0.153)	(0.179)
More than 65	0.291**	0.431***	0.197	0.341	0.132	0.606
	(0.114)	(0.134)	(0.125)	(0.260)	(0.124)	(0.482)
Secondary Education	0.112***	0.101**	0.144*	0.164***	0.106*	0.256***
	(0.039)	(0.040)	(0.075)	(0.050)	(0.056)	(0.091)
Higher Education	0.178***	0.146***	0.269***	0.333***	0.259***	0.392***
	(0.041)	(0.046)	(0.076)	(0.053)	(0.061)	(0.089)
Children	− 0.012	− 0.023*	0.015	− 0.018	0.001	− 0.031
	(0.013)	(0.013)	(0.030)	(0.026)	(0.029)	(0.054)
Round 2	0.055**	0.030	0.095**	0.099***	0.068*	0.145**
	(0.024)	(0.029)	(0.039)	(0.037)	(0.041)	(0.067)
Round 3	0.212***	0.233***	0.189***	0.268***	0.258***	0.270***
	(0.032)	(0.045)	(0.043)	(0.042)	(0.053)	(0.064)
Constant	− 0.117***	− 0.131**	− 0.139*	− 0.112	− 0.131	− 0.120
	(0.041)	(0.052)	(0.082)	(0.072)	(0.081)	(0.136)
Observations	3110	1569	1541	1854	946	908
R-squared	0.187	0.194	0.233	0.166	0.173	0.169

Coefficients are from linear models. Standard errors clustered at the regional level. Sampling weights applied. ****p* < 0.01, ***p* < 0.05, **p* < 0.1

A10, and

**Table A11:** Determinants of knowledge of someone with COVID-19, Nigeria

Variables	(1)	(2)	(3)	(4)	(5)	(6)
	Women	Rural women	Urban women	Men	Rural men	Urban men
Migrated within state from 2018 to 2019 to Round 1 location	0.047	− 0.129**	0.151	− 0.013	− 0.004	− 0.025
	(0.083)	(0.055)	(0.129)	(0.033)	(0.057)	(0.036)
Migrated to another state from 2018 to 2019 to Round 1 location	0.017	− 0.045	0.065	0.027	0.046	0.029
	(0.046)	(0.049)	(0.072)	(0.043)	(0.063)	(0.053)
Single	− 0.026	− 0.052	− 0.005	− 0.094***	− 0.008	− 0.145***
	(0.021)	(0.034)	(0.026)	(0.031)	(0.042)	(0.041)
Widowed	− 0.001	− 0.043	0.020	− 0.167***	− 0.046	−
	(0.081)	(0.044)	(0.129)	(0.040)	(0.042)	
Separated–Divorced	0.353*	0.074	0.395*	− 0.094**	− 0.037	0.118**
	(0.198)	(0.159)	(0.210)	(0.048)	(0.038)	(0.050)
Age 26–35	0.014	0.002	0.015	− 0.003	0.013	− 0.013
	(0.021)	(0.037)	(0.025)	(0.024)	(0.037)	(0.030)
Age 36–45	0.055	0.035	0.055	− 0.026	0.018	− 0.052
	(0.037)	(0.059)	(0.043)	(0.039)	(0.053)	(0.052)
Age 46–55	− 0.016	0.080	− 0.083**	− 0.022	− 0.039	− 0.028
	(0.050)	(0.082)	(0.036)	(0.050)	(0.061)	(0.067)
Age 56–65	0.129*	− 0.058	0.191*	− 0.083*	− 0.045	− 0.108*
	(0.070)	(0.036)	(0.103)	(0.047)	(0.059)	(0.063)
More than 65	0.353	0.066	0.457	0.402*	− 0.085*	0.481**
	(0.250)	(0.088)	(0.291)	(0.220)	(0.052)	(0.193)
Secondary Education	− 0.020	0.027	− 0.076	0.084**	0.100***	0.075
	(0.038)	(0.037)	(0.062)	(0.037)	(0.031)	(0.073)
Higher Education	0.078*	0.142**	0.024	0.140***	0.112***	0.144**
	(0.043)	(0.060)	(0.060)	(0.036)	(0.032)	(0.071)
Children	− 0.005	0.021*	− 0.016*	0.020**	0.022**	0.023*
	(0.008)	(0.013)	(0.009)	(0.009)	(0.010)	(0.014)
Round 2	0.016	− 0.006	0.020	− 0.017	− 0.018	− 0.015
	(0.013)	(0.018)	(0.017)	(0.018)	(0.027)	(0.025)
Round 3	0.049***	0.015	0.059**	0.061***	0.028	0.083***
	(0.018)	(0.026)	(0.024)	(0.021)	(0.033)	(0.029)
Constant	0.024	0.002	0.057	0.034	− 0.030	0.073
	(0.039)	(0.044)	(0.060)	(0.052)	(0.053)	(0.089)
Observations	2703	943	1760	2070	762	1308
R-squared	0.090	0.089	0.147	0.083	0.048	0.125

Coefficients are from linear models. Standard errors clustered at the regional level. Sampling weights applied. ****p* < 0.01, ***p* < 0.05, **p* < 0.1

A11.

## Footnotes

1 In Round 1, 4(7) Kenyan and 3(1) Nigerian respondents indicate migrating out of the country in 2019(2020).

2 The outcomes are defined as the following. The migrant outcome assigns a value of one for those who migrate within or across regions, and a value of zero for non-movers. The migrate within region outcome assigns a value of one for those who migrate within region, and a value of zero for non-movers and migrants who move across regions. The migrate to another region outcome assigns a value of one for those who migrate to another region, and a value of zero for non-movers and migrants who move within region.

3 The regression equation of the linear probability model is: ${M}_{it}={\alpha }_{0}+{\alpha }_{t}+\beta {X}_{i}+{\gamma }_{j}{\alpha }_{t}{X}_{i}+{\varepsilon }_{it},$ where *t* = {2019, 2020, 2021}*.* The dependent variable *M* indicates whether the individual *i* migrated in year t. Two additional regressions were evaluated using the dependent variable, whether the individual migrated within the county/state and whether the individual migrated to another county/state. *X* is a vector of baseline covariates, while ${\alpha }_{t}$ is the year fixed effect. All standard errors are clustered at the county level for Kenya and the state level for Nigeria. The coefficient of interest is ${\gamma }_{j}$. If ${\gamma }_{j}$ is statistically different than zero, then that specific demographic group might have experienced a change in their mobility patterns relative to 2019.

## Declarations

### Permission to Reproduce Material from Other Sources

N/A.
